# Next‐generation sequencing identifies rare pathogenic and novel candidate variants in a cohort of Chinese patients with syndromic or nonsyndromic hearing loss

**DOI:** 10.1002/mgg3.1539

**Published:** 2020-10-23

**Authors:** Yan‐Bao Xiang, Chen‐Yang Xu, Yun‐Zhi Xu, Huan‐Zheng Li, Li‐Li Zhou, Xue‐Qin Xu, Zi‐Hui Chen, Shao‐Hua Tang

**Affiliations:** ^1^ Key Laboratory of Birth Defects Department of Genetics Wenzhou Central Hospital Wenzhou China; ^2^ Key laboratory of Medical Genetic School of Laboratory Medicine and Life Science Wenzhou Medical University Wenzhou China

**Keywords:** hearing loss, molecular diagnosis, next‐generation sequencing

## Abstract

**Background:**

Hearing loss (HL) is a common sensory disorder in humans characterized by extreme clinical and genetic heterogeneity. In recent years, next‐generation sequencing (NGS) technologies have proven to be highly effective and powerful tools for population genetic studies of HL. Here, we analyzed clinical and molecular data from 21 Chinese deaf families who did not have hotspot mutations in the common deafness genes *GJB2*, *SLC26A4*, *GJB3*, and *MT*‐*RNR1*.

**Method:**

Targeted next‐generation sequencing (TGS) of 127 known deafness genes was performed in probands of 12 families, while whole‐exome sequencing (WES) or trio‐WES was used for the remaining nine families.

**Results:**

Potential pathogenic mutations in a total of 12 deafness genes were identified in 13 probands; the mutations were observed in *GJB2*, *CDH23*, *EDNRB*, *MYO15A*, *OTOA*, *OTOF*, *TBC1D24*, *SALL1*, *TMC1*, *TWNK*, *USH1C*, and *USH1G*, with eight of the identified mutations being novel. Further, a copy number variant (CNV) was detected in one proband with heterozygous deletion of chromosome 4p16.3‐4p15.32. Thus, the total diagnostic rate using NGS in our deafness patients reached 66.67% (14/21).

**Conclusions:**

These results expand the mutation spectrum of deafness‐causing genes and provide support for the use of NGS detection technologies for routine molecular diagnosis in Chinese deaf populations.

## Graphical abstract

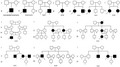

## 1 INTRODUCTION

Hearing loss (HL) is an extremely complex and heterogeneous disorder affecting nearly one in 300‐1000 infants (Morton & Nance, 2006; Morton, 1991). More than half of patients with HL have an identified underlying genetic cause, with the HL either occurring as an isolated condition (nonsyndromic; 70%) or presenting with additional systemic manifestations (syndromic; 30%; Sakuma et al., 2016; Smith et al., 2005). To date, around 110 genes and 150 loci have been found to be associated with HL (https://hereditaryhearingloss.org/). The genes most commonly detected in Chinese deaf populations are *GJB2* (121,011), *SLC26A4* (605,646), mtDNA *12SrRNA* (561,000), and *GJB3* (603,324), accounting for approximately 30‐50% of cases (Jiang et al., 2015; Ming et al., 2019; Xiang et al., 2019). For the remaining cases, deafness is attributable to rare mutations in identified deafness genes or unknown etiologies. Here, we enrolled 21 Chinese patients with either syndromic or nonsyndromic HL who were previously evaluated and were not found to have hotspot mutations in the common deafness genes *GJB2*, *SLC26A4*, *MT*‐*RNR1*, and *GJB3*. Next‐generation sequencing (NGS) technologies, including targeted NGS (TGS) and whole‐exome sequencing (WES), were performed on the probands of each family to identify rare pathogenic mutations. The findings of this study illustrate the genetic heterogeneity of HL and highlight the value of the NGS approach in patients with complex clinical phenotypes.

## 2 MATERIALS AND METHODS

### 2.1 Patients and clinical information

A total of 21 deaf families, including six syndromic hearing loss (SHL) families and 15 nonsyndromic hearing loss (NSHL) families, were recruited from Wenzhou Central Hospital from 2017 to 2020. Written informed consent for participation in the study was obtained from each patient or their guardians. This study was approved by the Ethics Committee of Wenzhou Central Hospital, Zhejiang, China. All patients had (a) bilateral HL, and (b) no hotspot mutations in common deafness genes, including *GJB2*, *SLC26A4*, *MT*‐*RNR1*, and *GJB3*. A comprehensive history and detailed physical examination records were obtained for each patient, including clinical history, infections, ototoxic drugs, possible head or brain injury, family history, and other clinical information related to the HL. Vestibular function and ophthalmologic evaluations were performed in two families (HL05 and HL20) with suspected Usher syndrome. Vestibular function was evaluated through statokinetic tests (Romberg, sensitized Romberg) and spontaneous nystagmus was detected by videonystagmography. The ophthalmologic evaluation included measurement of best‐corrected visual acuity, dilated fundus ophthalmoscopy, and optical coherence tomography (OCT). Pure‐tone audiometry (PTA) or auditory steady‐state evoked response (ASSR) and/or auditory brainstem response (ABR) were used to assess the degree and progression of HL in all probands. HL severity was classified as mild (26–40 dB), moderate (40–60 dB), severe (60–80 dB), or profound (>80 dB).

### 2.2 Prescreening of hotspot mutations in common deafness genes

Blood samples were obtained from the family members and genomic DNA was extracted from the whole blood, according to standard procedures, using a Qiagen DNA Blood Midi/Mini Kit (Qiagen). Prescreening of hotspot mutations in *GJB2* (c.35delG, c.176_191del16, c.235delC, c.299‐300delAT), *SLC26A4* (c.919‐2A>G, c.1174A>T, c.1226G>A, c.1229C>T, c.1707+5G>A, c.1975G>C, c.2027T>A, c.2168A>G), *MT*‐*RNR1* (m.1494C>T, m.1555A>G), and *GJB3* (c.538C>T) was performed in all probands by microarray (CapitalBio, China; Xiang et al., 2019). Of the blood samples collected from the probands of the 21 deaf families, 12 were analyzed by TGS of 127 deafness‐causing genes (Table S1), six were analyzed by single proband WES, and the remaining three were analyzed by trio‐WES (WES for family proband and parents simultaneously).

### 2.3 NGS and data analysis

For TGS, a custom human array was constructed using Roche NimbleGen, targeting exons and 10 bp flanking intronic sequences of a total of 127 HL‐causing genes; these HL‐causing genes were identified from four well‐known public databases (The Hereditary Hearing Loss Homepage, Deafness Variation Database, GeneReviews, and Orphanet). For WES, exon‐containing fragments and their splice junctions were enriched by SureSelect Human All Exon V6 (Agilent). DNA sequencing was performed with a HiSeq2000 sequencer (Illumina). Raw data generated by NGS were filtered to obtain high‐quality clean reads and was further aligned to the NCBI Human Reference Genome (hg19/GRCh37) using the Burrows‐Wheeler Aligner (BWA). SAMtools and GATK were applied for annotation of BAM files. Candidate pathogenic variants were defined as missense, nonsense, or frameshift mutations and splice sites with a frequency lower than 0.005, using gnomAD (http://gnomad.broadinstitute.org/), the 1000 Genomes Project database (http://browser.1000genomes.org), and NCBI dbSNP (http://www.ncbi.nlm.nih.gov/snp). The mutation databases Clinvar (http://www.ncbi.nlm.nih.gov/clinvar), HGMD (http://www.hgmd.org), and OMIM (http://omim.org/) were used to annotate previously reported pathogenic mutations for HL. The synonymous variants in the coding region and variants in the intronic (with the exception of the splice mutation that may create an ectopic site) or untranslated regions were filtered out. The pathogenicities of the nonsplicing site variants were predicted by a variety of computational tools including Polyphen‐2 (http://genetics.bwh.harvard.edu/pph2), SIFT (http://sift.jcvi.org), and Mutation Taster (http://www.mutationtaster.org). The pathogenicities of the splicing site variants were predicted by Human Splicing Finder (http://www.umd.be/HSF3/).

### 2.4 Confirmation and segregation analysis

The candidate pathogenic variants identified by data analysis were confirmed using direct Sanger sequencing on an ABI3130 DNA analyzer (Applied Biosystems). Segregation analysis was then performed for the proband's family members. Based on the guidelines of American College of Medical Genetics and Genomics (ACMG, http://www.acmg.net/), selected variants were classified as pathogenic, likely pathogenic, variants with uncertain significance (VUS), likely benign or benign (Richards et al., 2015).

## 3 RESULTS

### 3.1 Clinical features

**FIGURE 1:**
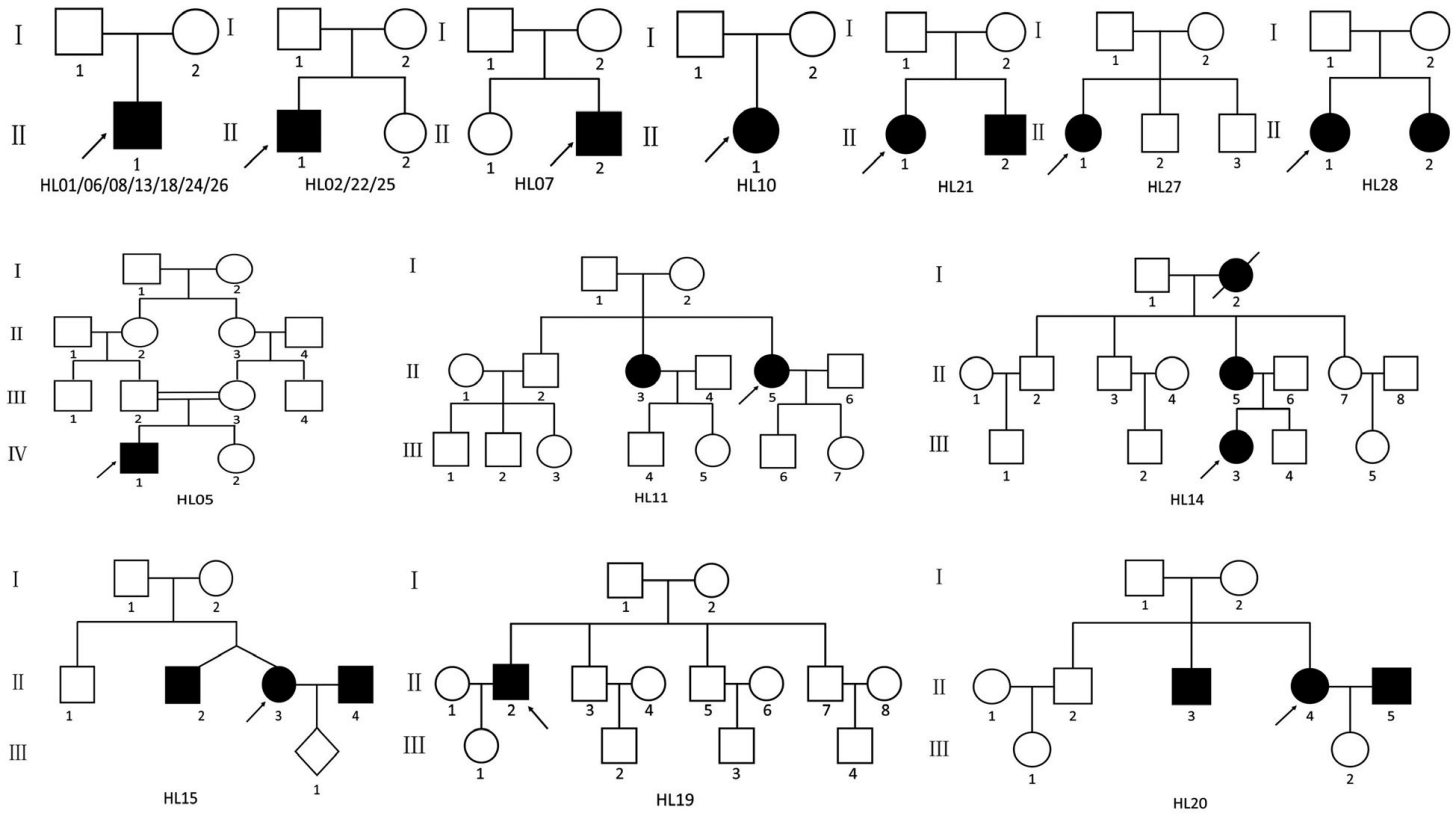
Pedigrees of 21 hearing loss families

A total of 21 HL families were enrolled in this study, including 15 simplex and six multiplex families (Figure 1). Patients in the 21 families ranged in age from 3 to 62 years and had variable onset ages from birth to 9 years. All patients had bilateral HL with variable developing course and degree of severity, ranging from stable to progressive and from mild to profound. Detailed physical examinations of the patients did not reveal any symptoms or malformations, aside from HL, in 15 cases. This suggests that, for these 15 patients, HL was nonsyndromic. However, multiple symptoms besides HL, such as retinitis pigmentosa (RP), vestibular dysfunction, developmental delay, spinocerebellar ataxia, dysplastic ears, and other systemic abnormalities, were detected in the remaining six families. Based on the clinical records and molecular diagnosis results, we categorized these six patients as Usher syndrome (2), Waardenburg syndrome (1), Townes‐Brocks syndrome (1), Perrault syndrome (1), and Wolf‐Hirschhorn syndrome (1). The clinical and instrumental features of the deaf probands are summarized in Table 1.

**Table 1:** Clinical features and detection methods of the 21 probands with syndromic or nonsyndromic hearing loss

Family ID	Age	Sex	Phenotype	Hearing loss		Inheritance	Clinical diagnosis	Detection methods
				Degree	Onset			
HL01	28 years	M	SNHL	Mild	Postlingual	AR	NSHL	127‐gene panel
HL02	8 years	M	SNHL	Profound	Congenital	AR	NSHL	127‐gene panel
HL05	37 years	M	SNHL, Retinitis pigmentosa, Vestibular defect	Profound	Congenital	AR	Usher syndrome type 1	WES
HL06	5 years	M	SNHL	Profound	Congenital	AR	NSHL	WES
HL07	25 years	M	SNHL	Severe	Congenital	AR	NSHL	WES
HL08	11 years	M	SNHL	Severe	Congenital	Unknown	NSHL	127‐gene panel
HL10	6 years	F	SNHL	Moderate‐severe	Congenital	Unknown	NSHL	127‐gene panel
HL11	62 years	F	SNHL	Profound	Congenital	AR	NSHL	127‐gene panel
HL13	5 years	M	SNHL	Profound	Congenital	AR	NSHL	WES
HL14	25 years	F	SNHL	Severe‐profound	Postlingual	Unknown	NSHL	WES
HL15	25 years	F	SNHL, Heterochromia iridis	Profound	Congenital	AR	Waardenburg syndrome type 4	WES
HL18	6 years	M	Mixed HL, Small left kidney, Bilaterally preaxial hexadactyly of the hands, Mild developmental delay, Low set of the ear, Dysplastic ears, Preauricular tags	Moderate‐profound	Congenital	AD	Townes‐Brocks syndrome 1	Trio‐WES
HL19	51 years	M	SNHL	Moderate‐severe	Congenital	Unknown	NSHL	127‐gene panel
HL20	43 years	F	SNHL, Retinitis pigmentosa	Profound	Congenital	AR	Usher syndrome type 1	127‐gene panel
HL21	8 years	F	SNHL, Spinocerebellar ataxia, Mild intellectual disability, Development delay	Moderate‐profound	Congenital	AR	Perrault syndrome type 5	Trio‐WES
HL22	26 years	M	SNHL	Severe	Congenital	AR	NSHL	127‐gene panel
HL24	3 years	M	SNHL, Intellectual disability, Development delay, Seizures, Cleft palate, Heart defects, Hypospadias, Micrognathia	Severe	Congenital	AD	Wolf‐Hirschhorn syndrome	Trio‐WES
HL25	28 years	M	SNHL	Profound	Congenital	Unknown	NSHL	127‐gene panel
HL26	27 years	M	SNHL	Profound	Congenital	AR	NSHL	127‐gene panel
HL27	28 years	F	SNHL	Profound	Congenital	Unknown	NSHL	127‐gene panel
HL28	25 years	F	SNHL	Severe	Congenital	Unknown	NSHL	127‐gene panel

Abbreviations: F, female; M, male; NSHL, nonsyndromic hearing loss; SNHL, sensorineural hearing loss; WES, whole‐exome sequencing.

### 3.2 Variant analysis and validation

The 21 HL family probands had been previously excluded from having 15 hotspot mutations in the *GJB2*, *SLC26A4*, *GJB3*, and *MT*‐*RNR1* genes according to microarray analysis. Thus, NGS was performed on all 21 probands, as requested by the patients themselves. The mean read depths of the targeted region for TGS and WES were 291.43× and 233.81×, respectively. The percentage of mappable bases representing a coverage of at least 30× for TGS and at least 20× for WES was 98.70% and 99.02%, respectively. To identify the potential causative mutations by NGS, variants with allele frequencies lower than 0.005 were filtered (except for the pathogenic mutations recorded in the HGMD, Clinvar, and OMIM databases). In total, pathogenic mutations were identified in 13 HL families and chromosome fragment deletion was detected in one family. Thus, the total diagnostic rate using NGS in our deafness patients reached 66.67% (14/21).

**FIGURE 2:**
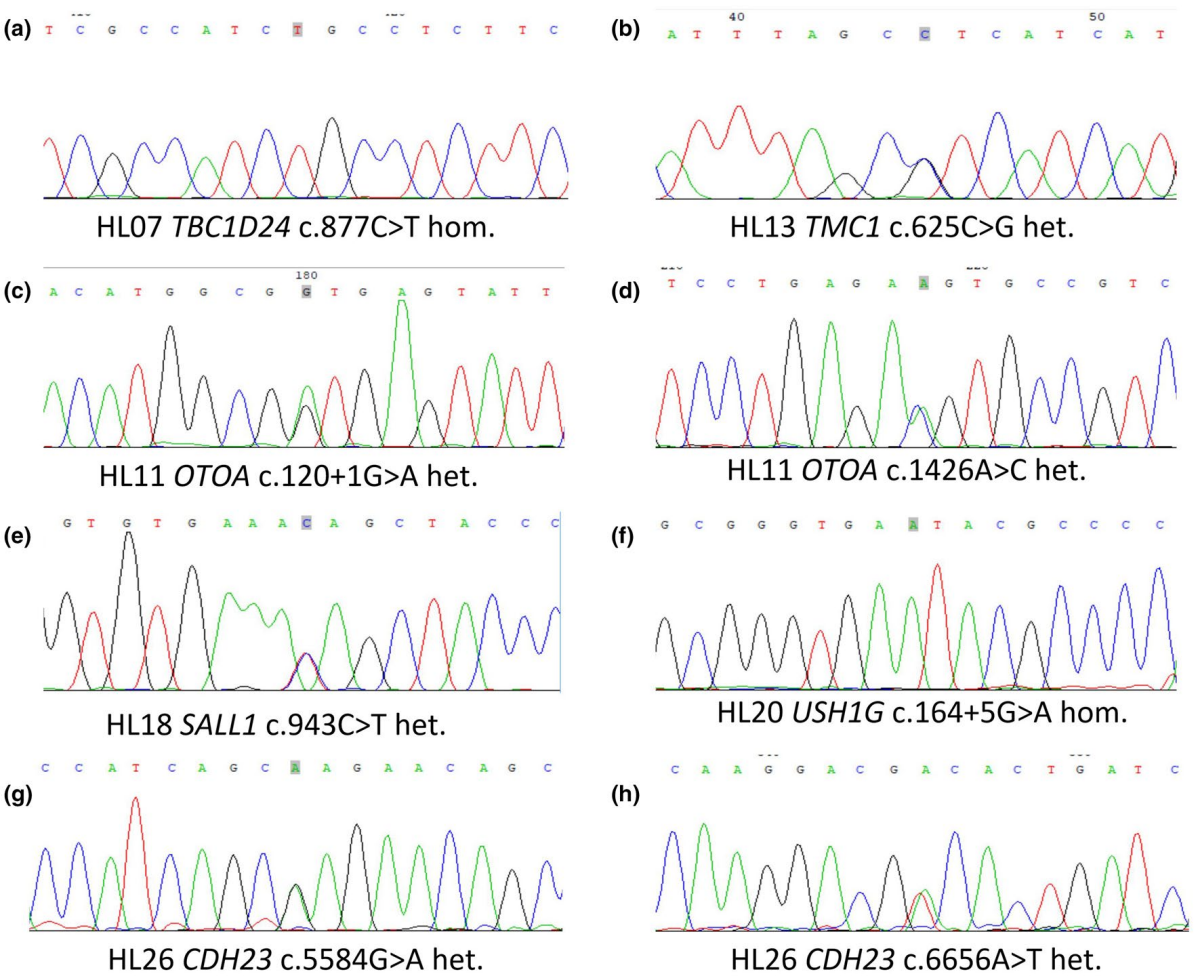
DNA sequencing chromatograms presenting eight novel mutations in rare deafness‐causing genes identified in six hearing loss families. (a) A homozygous missense mutation c.877C>T of *TBC1D24* in family HL07. (b) A heterozygous missense mutation c.625C>G of *TMC1* in family HL13. (c) A heterozygous splicing mutation c.120+1G>A of *OTOA* in family HL11. (d) A heterozygous missense mutation c.1426A>C of *OTOA* in family HL11. (e) A heterozygous nonsense mutation c.943C>T of *SALL1* in family HL18. (f) A homozygous missense mutation c.164+5G>A of *USH1G* in family HL20. (g) A heterozygous missense mutation c.5584G>A of *CDH23* in family HL26. (h) A heterozygous missense mutation c.6656A>T of *CDH23* in family HL26

Among the 14 patients with identified pathogenic factors (Table 2), 12 had homozygous or compound heterozygous mutations in deafness‐causing genes, including mutations in *GJB2* (HL01, HL22), *OTOF* (603,681; HL02), *USH1C* (605,242; HL05), *MYO15A* (602,666; HL06), *TBC1D24* (613,577; HL07), *OTOA* (607,038; HL11), *TMC1* (606,706; HL13), *EDNRB* (131,244; HL15), *USH1G* (607,696; HL20), *TWNK* (606,075; HL21), and *CDH23* (601,067; HL26), suggesting recessive genetic forms of HL. Further, the family HL18 proband was found to have a de novo heterozygous mutation of *SALL1* c.943C>T (p.Q315X), suggesting a dominant state of this mutation. Sanger sequencing in the probands and other family members confirmed the cosegregation of the candidate pathogenic mutations with the hearing clinical phenotype (Table S2). Among the 17 mutations identified in our cohort, eight have not been associated with deafness in previous reports (Figure 2). All eight novel mutations were predicted to be deleterious by at least one computational program. Further, a de novo CNV of 15.89 Mb deletion at chromosome 4p16.3p15.32 region (seq[hg19] 4p16.3‐4p15.32 (331773_16228080)×1) was identified in the family HL24 proband, which was considered to be the molecular basis of Wolf‐Hirschhorn syndrome.

**Table 2:** Mutations in deafness‐associated genes identified in 14 HL families

Family ID	Gene	Reference sequence	cDNA change	Amino acid change	Zygosity	gnomAD (All)	HSF	Polyphen−2 (Score)	SIFT (Score)	Mutation taster	Evidence criterion (ACMG)	References or Classification
HL01	*GJB2*	NM_004004	c.109G>A	p.V37I	Hom	/	/	/	/	/		Chen et al. (2016)
HL02	*OTOF*	NM_194248	c.4023+1G>A	Splicing	Het	/	/	/	/	/		Wang et al. (2011)
		NM_194248	c.5026C>T	p.R1676C	Het	/	/	/	/	/		Wang et al. (2011)
HL05	*USH1C*	NM_001297764	c.311G>A	p.G104D	Hom	/	/	/	/	/		Besnard et al. (2014)
HL06	*MYO15A*	NM_016239.4	c.9400C>T	p.R3134X	Hom	/	/	/	/	/		Schrauwen et al. (2013)
HL07	*TBC1D24*	NM_001199107.1	c.877C>T	p.R293C	Hom	0.000008	/	D (0.980)	D (0.001)	DC	PM2+PM5+PP1+PP3	Novel (LP)
HL11	*OTOA*	NM_144672	c.120+1G>A	Splicing	Het	0	P	/	/	/	PVS1+PM2+PP1	Novel (P)
		NM_144672	c.1426A>C	p.S476R	Het	/	/	D (0.642)	T (0.053)	PO	PM2+PM3+PP1+PP3	Novel (LP)
HL13	*TMC1*	NM_138691	c.625C>G	p.L209V	Het	/	/	D (0.989)	D (0.014)	DC	PM2+PM3+PP1+PP3	Novel (LP)
		NM_138691	c.2050G>C	p.D684H	Het	/	/	/	/	/		Jiang et al. (2018)
HL15	*EDNRB*	NM_000115	c.553G>A	p.V185M	Hom	/	/	/	/	/		Wang et al. (2017)
HL18	*SALL1*	NM_002968	c.943C>T	p.Q315X	Het	0.000004	/	/	/	DC	PVS1+PS2+PP1	Novel (P)
HL20	*USH1G*	NM_173477	c.164+5G>A	Splicing	Hom	0	P	/	/	/	PM2+PP1+PP3+PP4	Novel (VUS)
HL21	*TWNK*	NM_021830.4	c.1172G>A	p.R391H	Het	/	/	/	/	/		Morino et al. (2014)
		NM_021830.4	c.1844G>C	p.G615A	Het	/	/	/	/	/		Li et al. (2019)
HL22	*GJB2*	NM_004004	c.109G>A	p.V37I	Hom	/	/	/	/	/		Chen et al. (2016)
HL26	*CDH23*	NM_022124	c.5584G>A	p.E1862K	Het	0.000012	/	D (0.908)	D (0)	DC	PM2+PM3+PP1+PP3	Novel (LP)
		NM_022124	c.6656A>T	p.D2219V	Het	0.000004	/	D (0.985)	D (0.001)	DC	PM2+PM5+PP1+PP3	Novel (LP)
		NM_022124	c.9058C>T	p.R3020C	Het	0.000200	/	B (0.380)	D (0.005)	DC		Novel (LB)
HL24	/	/	15.89 Mb deletion of the 4p16.3p15.32 region		Het	/	/	/	/	/		Novel

Abbreviations: B, benign; D, deleterious or damaging; DC, disease causing; Het, heterozygous; Hom, homozygous; LB, likely benign; LP, likely pathogenic; P, pathogenic; PO, polymorphism; T, tolerated; VUS, Variants with uncertain significance.

## 4 DISCUSSION

In the present study, we performed comprehensive genetic testing using NGS technology in 21 nonsyndromic and syndromic Chinese Han deaf families. The results indicated that a total of 14 patients (66.7%; 14/21) with HL had clear molecular etiology, higher than the 15.8%–37.2% diagnostic rates for exome sequencing or HL gene panel analysis in previous reports (Chen et al., 2016;Likar et al., 2018; Sheppard et al., 2018; Zou et al., 2020). Our NGS analysis results demonstrate the effectiveness of NGS for routine diagnosis among deaf patients with NSHL or SHL.

### 4.1 Families with NSHL

A total of eight of the 15 NSHL families were identified to have pathogenic mutations in deafness‐causing genes. In families HL01 and HL22, homozygous mutation of *GJB2* c.109G>A (p.V37I) was identified by TGS in both probands. c.109G>A is a controversial mutation and was once reported to be a polymorphism variant (Kelly et al., 1998). However, recent studies have shown that c.109G>A is a pathogenic mutation that can cause extremely heterogeneous phenotypes of HL, from mild to profound, with HL onset occurring congenitally to during adulthood (Chen et al., 2016; Shen et al., 2017). This is in agreement with our clinical records and audiological testing results for these two probands; HL01‐Ⅱ1 had nonprogressive mild hearing impairment with onset around 3 years of age, whereas individual HL22‐Ⅱ1 exhibited a more severe clinical phenotype with severe sensorineural HL at birth. Thus, our results add support for the pathogenicity of the c.109G>A mutation, which can lead to various HL phenotypes. These results indicate that c.109G>A should be considered to be a routine hotspot mutation for screening of *GJB2* in NSHL patients in China.

In family HL02, TGS revealed a compound heterozygous mutation of c.4023+1G>A/c.5026C>T (p.Arg1676Cys) in *OTOF*, a gene associated with auditory neuropathy spectrum disorder (ANSD; Bai et al., 2019; Wang et al., 2011). ANSD is a subtype of sensorineural hearing loss (SNHL) characterized by a nonevoked ABR and normal response otoacoustic emissions (OAEs). Similar to most *OTOF* cases in the literature that present with profound NSHL, our affected individual, HL02‐Ⅱ1, had profound congenital HL and the ABR was absent (OAEs were not examined). Both c.4023+1G>A and c.5026C>T mutations have been reported in ANSD patients by Wang et al. (2011); however, the pathogenicity of these two mutations is unclear. The recessive inheritance and verification of DNA sequences in family members (including unaffected parents and sibling) confirmed that these two mutations in *OTOF* cosegregated with the HL, demonstrating the pathogenicity of these two mutations.

Mutations in *MYO15A* are frequently detected in patients with severe to profound SNHL, and *MYO15A* is considered to be one of the most common deafness‐causing genes responsible for NSHL in Chinese patients (Bai et al., 2019; Wang et al., 2011; Yang et al., 2013). In family HL06, the affected individual, HL06‐Ⅱ1, was a 5‐year‐old boy and was found to have a homozygous mutation of *MYO15A* c.9400C>T (p.R3134X; Schrauwen et al., 2013). The patient failed the newborn hearing screening test of the automated auditory brainstem response (AABR) and had congenital profound SNHL confirmed by ABR and ASSR at 2 years of age. The patient then received a cochlear implant and now exhibits good speech and language recognition.

Mutations in *TBC1D24* are frequently reported in patients with DOOR (Deafness, Onychodystrophy, Osteodystrophy, and mental Retardation) syndrome, myoclonic epilepsy, and epileptic encephalopathy; rarely are such mutations observed in patients with NSHL, including autosomal dominant deafness (DFNA65) and autosomal recessive deafness (DFNB86; Balestrini et al., 2016; Rehman et al., 2014; Zhang et al., 2014). Here, we identified a novel homozygous mutation of c.877C>T (p.R293C) by WES in the proband of family HL07. Sanger sequencing verified this mutation and showed a heterozygous mutation of c.877C>T in both of his unaffected parents and elder sister, suggesting that c.877C>T is cosegregated with HL in this family. The patient, HL07‐Ⅱ2, was a 25‐year‐old male. Similar to most *TBC1D24* mutations in DFNB86 cases with severe to profound sensorineural hearing impairment, our patient also presented with congenital severe hearing impairment. Thus, it is highly recommended that patients with severe or profound clinical phenotypes of HL are screened for mutations in *TBC1D24*.

In family HL11, two novel mutations c.120+1G>A/c.1426A>C(p.S476R) were identified in *OTOA*, which encodes Otoancorin and is required for the development of the tectorial membrane in the inner ear; *OTOA* is considered to be the molecular basis responsible for prelingual onset of moderate to profound sensorineural HL in some patients (Kim et al., 2019; Sugiyama et al., 2019). The two affected siblings, HL11‐Ⅱ3 and HL11‐Ⅱ5, were aged 64 and 62 years, respectively. Both patients experienced congenital profound sensorineural HL at all frequencies. Sanger sequencing verified the two mutations and suggested that both c.120+1G>A and c.1426A>C were segregated with HL in this family.

Mutations in *TMC1* can cause both autosomal dominant and recessive NSHL (Jiang et al., 2018; Kitajiri et al., 2007). We identified compound heterozygous mutations of c.625C>G (p.L209V) and c.2050G>C (p.D684H) in *TMC1* in family HL13, which were segregated with HL in this family. This suggests recessive inheritance of these two mutations. c.625C>G is a novel mutation, whereas c.2050G>C is a known mutation that is frequently detected in the deaf population in Xiamen, China (Jiang et al., 2018). Thus, c.2050G>C should be considered as a potential hotspot mutation in *TMC1* in the deaf population from China. Proband HL13‐Ⅱ1 passed the newborn hearing screening of the AABR but had profound sensorineural HL confirmed by ASSR at 10 months of age. At the age of 2 years, the patient received a right cochlear implant. Now, at age 6, he has good speech and language recognition.

Mutation in *CDH23* can lead to both NSHL (DFNB12) and Usher syndrome, characterized by congenital HL and RP (Moteki et al., 2016; Okano et al., 2019; Wu et al., 2020). Interestingly, three novel missense variants of c.5584G>A (p.E1862K), c.6656A>T (p.D2219V), and c.9058C>T (p.R3020C) were identified in *CDH23* in the family HL26 proband. However, only two of these were considered to be pathogenic. The patient was a 27‐year‐old male with congenital profound sensorineural HL. No other systemic abnormalities were detected. Sanger sequencing confirmed all three mutations and showed that c.5584G>A was inherited from his healthy father, while both c.6656A>T and c.9058C>T were inherited from his healthy mother. Given that c.6656A>T (p.D2219V) shares an identical amino acid position at p.D2219 with a known pathogenic mutation, c.6655G>A(p.D2219Q) (Moteki et al., 2016), and was predicted to be a pathogenic mutation by Polyphen‐2, SIFT, and Mutation Taster, it is most likely that c.6656A>T is the true pathogenic mutation, rather than c.9058C>T. Thus, biallelic mutations of c.5584G>A and c.6655G>A in *CDH23* are the potential genetic basis for the HL in this family.

### 4.2 Families with SHL

A total of six SHL families were investigated in this study; a 100% genetic variation diagnostic rate was obtained among these probands.

Mutation in *USH1C* and *USH1G* may lead to Usher syndrome type Ⅰ (USH1), characterized by congenital profound HL, vestibular dysfunction, and prepubertal onset of RP, eventually leading to legal blindness (Besnard et al., 2014; D’Esposito et al., 2019). Proband HL05‐Ⅳ1 was a male patient, aged 37 years at the time of our examination, born to healthy consanguineous (first cousin) parents. He presented with a typical USH1 phenotype of congenital profound HL, vestibular dysfunction, and onset of visual impairment at age 5 years. WES identified a previously reported homozygous mutation of c.311G>A (p.G104D) in *USH1C* in proband HL05‐Ⅳ1. Sanger sequencing verified this mutation and showed that both his parents and younger brother had heterozygous mutations of c.311G>A, suggesting that c.311G>A was cosegregated with Usher syndrome in this family. However, an atypical phenotype of USH1 was evident in proband HL20‐Ⅱ4 and her affected brother HL20‐Ⅱ3. In family HL20, the proband was a 43‐year‐old female with onset of nyctalopia at age 25 and her affected brother was a 45‐year‐old male with onset of nyctalopia at age 22. While HL was confirmed to be congenital profound, the proband had normal vestibular function. Although typical RP was present, the age at onset was remarkably late for USH1. TGS identified a novel homozygous splice mutation of c.164+5G>A in the *USH1G* gene in proband HL20‐Ⅱ4. Sanger sequencing verified this mutation and detected an identical homozygous mutation in her affected brother, HL20‐Ⅱ3. Heterozygous mutation of c.164+5G>A was also detected in both her mother, her daughter, and her eldest healthy brother, suggesting that this mutation was cosegregated with HL in this family. Given the consistent clinical phenotype of Usher syndrome, the low frequency in the public control population, and the familial cosegregation of *USH1G* c.164+5G>A, this variant was considered to be a potential pathogenic factor in family HL20. However, c.164+5G>A in *USH1G* is a noncanonical splice site variant and is only classified with VUS by ACMG; thus, whether it is truly pathogenic requires further study. *USH1G* classically causes USH1, but our patient exhibited a phenotype more consistent with Usher syndrome type Ⅱ(USH2, characterized by congenital moderate to severe HL, onset of RP in the first or second decade of life, normal vestibular function). Thus, our result may widens the spectrum of *USH1G*‐related allelic disorders.

*EDNRB* has been identified as a disease‐causing gene for Waardenburg syndrome type Ⅳ (WS4) and Hirschsprung disease (Wang et al., 2017). In family HL15, the proband, HL15‐Ⅱ3, and her twin brother, HL15‐Ⅱ2, were both 25 years old with identical phenotypes of congenital profound sensorineural HL and heterochromia iridis, with a suspected diagnosis of Waardenburg syndrome. Although a homozygous mutation of c.553G>A(p.V185M) (Wang et al., 2017) in *EDNRB* was identified by WES in this family, no other characteristic symptoms, such as Hirschsprung disease and depigmented patches of the skin and hair, were found in our patients, suggesting atypical WS4 disease. Sanger sequencing confirmed this mutation in the proband and showed an identical homozygous mutation in her twin brother and a heterozygous mutation of c.553G>A in both her normal parents and eldest brother, suggesting that c.553G>A was cosegregated with WS4. Heterozygous *EDNRB* c.553G>A mutation has been reported previously as a dominant pathogenic mutation in Chinese patients with Hirschsprung disease (PMID: 16944573). However, no consistent symptoms were observed in the proband's healthy parents and eldest brother, who carried a heterozygous mutation of c.553G>A; this suggests incomplete penetrance of this mutation in dominant inheritance in Hirschsprung disease. Further, the homozygous status of *EDNRB* c.553G>A in our patients may indicate a different form of recessive inheritance of this mutation in WS4 disease.

Townes‐Brocks syndrome (TBS) is a rare autosomal dominant disorder characterized by anal, hand, foot, and ear abnormalities; it has an estimated prevalence of 1:250,000 live births (Liberalesso et al., 2017). Here, we identified a previously unreported de novo nonsense mutation of *SALL1* c.943C>T (p.Q315X) in proband HL18‐Ⅱ1 by trio‐WES. The patient was a 6‐year‐old boy who was born with bilateral preaxial hexadactyly of the hands, dysplastic ear, low set left ear, bilateral preauricular tags, small left kidney, and hearing impairment. Moderate to profound mixed (sensorineural and conductive) HL was confirmed by audiological testing of ASSR, PTA, and ABR at age 4 years. Further, mild developmental delay and epicanthus were also evident in this proband. However, the most common feature of TBS, anal abnormality, was absent, demonstrating the high clinical heterogeneity of TBS.

Perrault syndrome 5 (PRLTS) is an autosomal recessive disorder characterized by sensorineural HL, female hypogonadotropic hypogonadism, and neurologic symptoms including ataxia, sensory neuropathy, nystagmus, muscle weakness, ophthalmoplegia, and intellectual disability (Li et al., 2019; Morino et al., 2014). In family HL21, we identified a biallelic mutation of c.1172G>A (p.R391H)/c.1844G>C (p.G615A) in *TWNK* in proband HL21‐Ⅱ1 and her affected younger brother HL21‐Ⅱ2. Both c.1172G>A (Morino et al., 2014) and c.1844G>C (Li et al., 2019) are known pathogenic mutations, and were inherited from the proband's father and mother, respectively. The two patients, aged 8 and 6 years at the time of evaluation, shared identical symptoms of progressive sensorineural HL from moderate to profound with onset at around 4 years of age. Ataxia, muscle hypotonia, and mild intellectual disability (IQ 62 and IQ 72 in proband HL21‐Ⅱ1 and her affected brother HL21‐Ⅱ2, respectively) were also present. Ophthalmoplegia was the only differential phenotype that was present in HL21‐Ⅱ2 but not in the proband.

Further, we detected a microdeletion syndrome associated with HL in family HL24 by trio‐WES. The patient, HL24‐Ⅱ1, was a 3‐year‐old boy who exhibited a series of symptoms, including micrognathia, cleft palate, developmental delay, mild intellectual disability, seizures, atrial septal defect, hypospadias, and mixed HL from moderate to severe. Data analysis indicated that the patient had Wolf‐Hirschhorn syndrome (WHS) with a de novo CNV of 15.89 Mb deletion at chromosome 4p16.3‐4p15.32 region (arr[hg19] 4p16.3‐4p15.32 (331773‐16228080)×1), according to the international system for cytogenetic nomenclature (ISCN) (McGowan‐Jordan et al., 2016). HL, an additional feature that is commonly present in WHS, can be detected in over 40% of WHS patients (Battaglia et al., 2015). Previous reports have shown that WHSC1 (NSD2)‐deficient mice display craniofacial abnormalities that overlap with WHS, including cochlea anomalies, suggesting that WHSC1 is a potential candidate gene associated with the HL phenotype (Ahmed et al., 2015). However, the pathogenesis of HL in WHS patients has not yet been clearly established.

## 5 CONCLUSION

In the present study, we examined clinical and molecular data from 21 Chinese deaf families and molecular diagnosis was made in 14 of 21 families. The results identified a number of novel and previously reported mutations in rare deafness‐causing genes. These findings highlight the importance of combining detailed clinical evaluation with NGS in the genetic diagnosis of NSHL and SHL patients. Moreover, the availability of precise molecular data in the very early stage of the disease may contribute to better monitoring of the disease itself and may help to improve the management of individual treatment strategies, such as cochlear implantation and early speech therapy.

## CONFLICT OF INTEREST

The authors declare that they have no competing interests.

## AUTHOR CONTRIBUTIONS

Y.B. Xiang and S.H. Tang defined the research theme. Y.B. Xiang, Y.Z. Xu, H.Z. Li, L.L. Zhou, and Z.H. Chen performed the experimental work and organized the data. S.H. Tang and X.Q. Xu are responsible for genetic counseling for the HL patient. Y.B. Xiang and C.Y. Xu designed the experiments and drafted the manuscript. All the authors read and approved the final manuscript.

## Supporting information

Table S1

Table S2
