# Supplementary material for: Next‐generation sequencing identifies rare pathogenic and novel candidate variants in a cohort of Chinese patients with syndromic or nonsyndromic hearing loss
Source: Mol Genet Genomic Med. 2020 Oct 23;8(12):e1539. doi: 10.1002/mgg3.1539 (PMC7767562; doi:10.1002/mgg3.1539)
Supplement: Supplementary file 1 — Table S1 [file MGG3-8-e1539-s001.docx]

**Supplementary material file 1:** Table S1. 127 genes targeted for next-generation sequencing

| *ALMS1*^S^ | *ACTG1*^N^ | *ATP2B2*^N^ | *BSND*^N/S^ | *CACNA1D*^S^ | *CCDC50*^N^ | *CDH23*^N/S^ |
| --- | --- | --- | --- | --- | --- | --- |
| CHD7^S^ | *CEACAM16*^N^ | *CLRN1*^S^ | *CLDN14*^N^ | *COCH*^N^ | *COL11A1*^S^ | *COL2A1*^S^ |
| *COL4A3*^S^ | *COL4A4*^S^ | *COL4A5*^S^ | *COL9A1*^S^ | *COLPA2*^S^ | *CRYM*^N^ | *DFNA5*^N^ |
| *DFNB31*^N/S^ | *DFNB59*^N^ | *DIABLO*^N^ | *DIAPH1*^N^ | *DIAPH3*^N^ | *DLX5*^S^ | *DSPP*^N^ |
| *EDN3*^S^ | *EDNRB*^S^ | *ESPN*^N^ | *ESRRB*^N^ | *EYA1*^S^ | *EYA4*^N^ | *FGF3*^S^ |
| *FGFR1*^S^ | *FGFR2*^S^ | *FGFR3*^S^ | *FOXI1*^N/S^ | *GATA3*^S^ | *GIPC3*^N^ | *GJA1*^N^ |
| *GJB2*^N^ | *GJB3*^N^ | *GJB6*^N^ | *GLI3*^S^ | *GPR98*^S^ | *GPSM2*^N^ | *GRHL2*^N^ |
| *GRXCR1*^N^ | *HGF*^N^ | *HOXA1*^S^ | *HOXA2*^S^ | *IGF1*^S^ | *ILDR1*^N^ | *KCNE1*^S^ |
| *KCNJ10*^N/S^ | *KCNQ1*^S^ | *KCNQ4*^N^ | *LHFPL5*^N^ | *LOXHD1*^N^ | *LRP2*^S^ | *LRTOMT*^N^ |
| *MARVELD2*^N^ | *MIR96*^N^ | *MITF*^S^ | *MSRB3*^N^ | *MT-RNR1*^N^ | *MT-TE*^S^ | *MT-TK*^S^ |
| *MT-TL1*^S^ | *MT-TS1*^N^ | *MYH14*^N^ | *MYH9*^N^ | *MYO15A*^N^ | *MYO1A*^N^ | *MYO3A*^N^ |
| *MYO6*^N^ | *MYO7A*^N/S^ | *NDP*^S^ | *OPA1*^S^ | *OTOA*^N^ | *OTOF*^N^ | *OTOG*^N^ |
| *PAX2*^S^ | *PAX3*^S^ | *PCDH15*^N/S^ | *PDSS1*^S^ | *PDZD7*^S^ | *PHEX*^S^ | *POU3F4*^N^ |
| *POU4F3*^N^ | *PRPS1*^N^ | *PRRX1*^N^ | *PTPRQ*^N^ | *RDX*^N^ | *SEMA3E*^S^ | *SERAC1*^S^ |
| *SERPINB6*^N^ | *SIX1*^N/S^ | *SIX5*^S^ | *SLC17A8*^N^ | *SLC19A2*^S^ | *SLC26A4*^N/S^ | *SLC26A5*^N^ |
| *SLC4A11*^S^ | *SMAD4*^S^ | *SMPX*^N^ | *SNAI2*^S^ | *SOBP*^S^ | *SOX10*^S^ | *SOX9*^S^ |
| *STRC*^N^ | *COF1*^S^ | *TECTA*^N^ | *TIMM8A*^S^ | *TJP2*^N^ | *TMC1*^N^ | *TMIE*^N^ |
| *TMPRSS3*^N^ | *TNFRSF11B*^S^ | *TPRN*^N^ | *TRIOBP*^N^ | *USH1C*^N/S^ | *USH1G*^S^ | *USH2A*^S^ |
| *WFS1*^N/S^ |  |  |  |  |  |  |

^N^Genes for non-syndromic hearing loss; ^s^Genes for syndromic hearing loss
