# Supplementary material for: Next‐generation sequencing identifies rare pathogenic and novel candidate variants in a cohort of Chinese patients with syndromic or nonsyndromic hearing loss
Source: Mol Genet Genomic Med. 2020 Oct 23;8(12):e1539. doi: 10.1002/mgg3.1539 (PMC7767562; doi:10.1002/mgg3.1539)
Supplement: Supplementary file 2 — Table S2 [file MGG3-8-e1539-s002.docx]

**Supplementary material file 2:** Table S2. Genotypes of family members from 14 hearing loss families.

| Family ID （Gene） | Member ID | cDNA change | Zygosity |
| --- | --- | --- | --- |
| HL01 （*GJB2*） | Ⅰ1 | c.109G>A | Heterozygous |
|  | Ⅰ2 | c.109G>A | Heterozygous |
|  | Ⅱ1 | c.109G>A | Homozygous |
| HL02 （*OTOF*） | Ⅰ1 | c.5026C>T | Heterozygous |
|  | Ⅰ2 | c.4023+1G>A | Heterozygous |
|  | Ⅱ1 | c.4023+1G>A/c.5026C>T | Compound heterozygous |
|  | Ⅱ2 | c.5026C>T | Heterozygous |
| HL05 （*USH1C*） | Ⅲ2 | c.311G>A | Heterozygous |
|  | Ⅲ3 | c.311G>A | Heterozygous |
|  | Ⅳ1 | c.311G>A | Homozygous |
|  | Ⅳ2 | c.311G>A | Heterozygous |
| HL06 （*MYO15A*） | Ⅰ1 | c.9400C>T | Heterozygous |
|  | Ⅰ2 | c.9400C>T | Heterozygous |
|  | Ⅱ1 | c.9400C>T | Homozygous |
| HL07 （*TBC1D24*） | Ⅰ1 | c.877C>T | Heterozygous |
|  | Ⅰ2 | c.877C>T | Heterozygous |
|  | Ⅱ1 | c.877C>T | Heterozygous |
|  | Ⅱ2 | c.877C>T | Homozygous |
| HL11 （*OTOA*） | Ⅱ2 | / | Wildtype |
|  | Ⅱ3 | c.120+1G>A/c.1426A>C | Compound heterozygous |
|  | Ⅱ5 | c.120+1G>A/c.1426A>C | Compound heterozygous |
|  | Ⅲ6 | c.1426A>C | Heterozygous |
|  | Ⅲ7 | c.120+1G>A | Heterozygous |
| HL13 （*TMC1*） | Ⅰ1 | c.2050G>C | Heterozygous |
|  | Ⅰ2 | c.625C>G | Heterozygous |
|  | Ⅱ1 | c.625C>G/ c.2050G>C | Compound heterozygous |
| HL15 （*EDNRB*） | Ⅰ1 | c.553G>A | Heterozygous |
|  | Ⅰ2 | c.553G>A | Heterozygous |
|  | Ⅱ1 | c.553G>A | Heterozygous |
|  | Ⅱ2 | c.553G>A | Homozygous |
|  | Ⅱ3 | c.553G>A | Homozygous |
| HL18 （*SALL1*） | Ⅰ1 | / | Wildtype |
|  | Ⅰ2 | / | Wildtype |
|  | Ⅱ1 | c.943C>T | Heterozygous |
| HL20（*USH1G*） | Ⅰ2 | c.164+5G>A | Heterozygous |
|  | Ⅱ2 | c.164+5G>A | Heterozygous |
|  | Ⅱ3 | c.164+5G>A | Homozygous |
|  | Ⅱ4 | c.164+5G>A | Homozygous |
|  | Ⅱ5 | / | Wildtype |
|  | Ⅲ2 | c.164+5G>A | Heterozygous |
| HL21 （*TWNK*） | Ⅰ1 | c.1172G>A | Heterozygous |
|  | Ⅰ2 | c.1844G>C | Heterozygous |
|  | Ⅱ1 | c.1172G>A/c.1844G>C | Compound heterozygous |
|  | Ⅱ2 | c.1172G>A/c.1844G>C | Compound heterozygous |
| HL22 （*GJB2*） | Ⅰ1 | c.109G>A | Heterozygous |
|  | Ⅰ2 | c.109G>A | Heterozygous |
|  | Ⅱ1 | c.109G>A | Homozygous |
|  | Ⅱ2 | c.109G>A | Heterozygous |
| HL26 （*CDH23*） | Ⅰ1 | c.5584G>A | Heterozygous |
|  | Ⅰ2 | c.6656A>T/c.9058C>T | Compound heterozygous |
|  | Ⅱ1 | c.5584G>A/c.6656A>T/c.9058C>T | Compound heterozygous |
| HL24 （*SALL1*） | Ⅰ1 | / | Wildtype |
|  | Ⅰ2 | / | Wildtype |
|  | Ⅱ1 | 15.89Mb deletion of the 4p16.3p15.32 region | Heterozygous |
